# How Family-Work Conflict Influences Post-traumatic Growth Among Medical Workers: A Moderated Mediation Model

**DOI:** 10.3389/fpsyg.2021.743970

**Published:** 2021-10-15

**Authors:** Miao Lv, Xuyun Tan, Cai Xing, Jiaren Zheng, Sixuan Han

**Affiliations:** ^1^School of Psychology, Qufu Normal University, Qufu, China; ^2^Institute of Sociology, Chinese Academy of Social Sciences, Beijing, China; ^3^Department of Psychology, Renmin University of China, Beijing, China; ^4^The Third Hospital of Jinjiang, Jinjiang, China; ^5^Department of Philosophy, Peking University, Beijing, China

**Keywords:** family-work conflict, post-traumatic growth, positive psychological capital, perceived social support, suppression, moderated mediation effect

## Abstract

Under the impact of COVID-19, the status and mechanisms of post-traumatic growth among medical workers facing challenges related to family-work conflict are of great concern. In view of the complex relationship between family-work conflict and post-traumatic growth, the present study sought to explore the specific relationships between family-work conflict and post-traumatic growth as well as the specific roles of positive psychological capital, perceived social support, and suppression. We recruited 1,347 participants. The results revealed that positive psychological capital and perceived social support played mediating roles, while suppression strategies moderated the mediating effect. Compared with the low suppression group, the negative impact of family-work conflict on positive psychological capital and perceived social support was reduced in the high suppression group. Thus, a higher level of suppression was more conducive to post-traumatic growth. The current study enriches and expands the findings of previous studies in theory and provides practical ways to promote post-traumatic growth in medical workers.

## Introduction

COVID-19 has severely impacted social and economic development, as well as individuals' work and family lives. As the COVID-19 pandemic continues to develop, medical workers on the frontline face increasing difficulties to balance their daily working and family lives. Moreover, frontline medical workers live with the constant threat of death and often witness many deaths. This situation can be difficult to change over a long period of time, and post-traumatic growth among workers may be particularly inhibited in the context of conflict, stress, and trauma. The relationships between family-work conflict and post-traumatic growth, the results have not been consistent, and the specific underlying mechanisms remain unclear. Therefore, the current study sought to examine the impact of family-work conflicts on post-traumatic growth and explore the mediating effect of positive psychological capital and perceived social support in this process, as well as explore the impact of emotional regulation strategies on this process.

### Impact of Family-Work Conflict on Post-traumatic Growth

Family-work conflict is a role conflict in which family and work pressures are in some way incompatible (Netemeyer et al., 1996), manifesting as conflict between fulfilling family responsibilities and the overall requirements, time-investment, and pressures of work. Family-work conflict includes both the time conflicts involved in caring for the family and completing work tasks, and the pressure of family roles hindering the completion of work responsibilities (Vaziri et al., 2020). The COVID-19 pandemic has caused changes in working status for many individuals, significantly exacerbating the conflict between family and work in some cases, and it has also directly affected individuals' mental health. One previous study reported that family-work conflict was a significant factor contributing to poor health in women (López-Núez et al., 2021). While many medical workers have made outstanding contributions in the fight against pandemic, workers have also made great sacrifices regarding family care. The pressure of family-work conflict during continuing pandemic, and interference with the working status of medical workers, increases the dual physical and psychological risks, and increases the likelihood of psychological trauma such as anxiety, obsessive-compulsive disorder, depression, and panic (Kang et al., 2020; Zhang et al., 2020).

Post-traumatic growth refers to positive psychological changes experienced after overcoming a challenging life crisis (Tedeschi and Calhoun, 1995). Individuals who experience traumatic events (e.g., earthquakes, cancer, pandemics) often exhibit not only negative psychological responses, but also positive changes in their appreciation of life, personal strength, relationships with others, spirituality, and outlook on new possibilities (Tedeschi and Calhoun, 1996).

COVID-19 is a negative life event for medical workers which can trigger traumatic experiences, and family-work conflict can strengthen the impact of negative life events, and inhibit the occurrence of growth (Netemeyer et al., 1996; Tedeschi and Calhoun, 2004). Many medical workers have experienced immense pressure and uncertainty during the COVID-19 pandemic, as well as psychological trauma related to the risk of death, which has caused some medical workers to undergo a process of self-reconstruction (Tedeschi and Calhoun, 2004). Previous studies have reported that the realization of post-traumatic growth is affected by many factors, including individual assessment control, helplessness, availability of personal resources, and transactional stress (Sheikh, 2008). Family-work conflicts can consistently induce these factors, such as feelings of helplessness and transactional pressure (Vaziri et al., 2020), which can restrain post-traumatic growth. Therefore, we proposed Hypothesis 1: Under the background of normal pandemic prevention and control, medical workers' long-term family-work conflicts will hinder the realization and improvement of post-traumatic growth.

### Family-Work Conflict and Post-traumatic Growth: The Mediating Roles of Perceived Social Support and Positive Psychological Capital

The job demands-resources (J-DR) model and conservation of resource (COR) theory argue that work requirements, including family-work conflict, are closely negatively related to work resources such as perceived social support and positive psychological capital (Bakker and Demerouti, 2017), while perceived social support and positive psychological capital are positively related to positive factors under pressure (Hobfoll, 1989). Thus, we speculate that the effects of family-work conflict on post-traumatic growth are likely to be achieved through perceived social support and positive psychological capital.

Perceived social support is the perceived availability of resources, including support provided by individual social networks, such as support from spouses, colleagues, friends, and family (Cohen and Wills, 1985). Family-work conflict is negatively related to perceived social support, which can help individuals reduce stress and adopt positive behaviors to cope with stress (Cohen and Wills, 1985). In the process of post-traumatic growth and improvement, individuals typically integrate positive changes, such as enhancing personal and social resources, independence, and efficiency into their lives (Tedeschi et al., 1998). According to the model of post-traumatic growth proposed by Tedeschi, perceived social support can help individuals with trauma to view things from different, more helpful, and adaptive perspectives and to develop new schemas (Tedeschi and Calhoun, 2004). Similarly, in the conceptual model proposed by Schaefer and Moos, perceived social support is regarded as a critical environmental resource used to understand the positive outcomes of life crises and transformation (Schaefer and Moos, 1998).

Positive psychological capital refers to a psychological state of individual positive development, which is a relatively new personality construction in positive organizational behavior, mainly manifested in self-efficacy, hope, optimism, and resilience (Luthans et al., 2007). In a study of nurses, the results revealed a significant negative correlation between family work conflict and resilience (Chen et al., 2021). In stressful situations, optimism, resilience, self-efficacy, and hope are all strategies that can help traumatized individuals recover from stress and achieve positive changes (Shand et al., 2015). In particular, hope about introducing expectations and goals for the future can help individuals shift their attention from practical difficulties and fear of negative outcomes to problem-solving behavior (Reff et al., 2005), exerting a direct positive impact on post-traumatic growth (Casellas-Grau et al., 2017).

At the same time, perceived social support as a perceived external support resource, and positive psychological capital as an internal positive resource, can improve the post-traumatic growth of individuals. The positive effects of both factors on post-traumatic growth can be explained by adaptive strategies (Zoellner and Maercker, 2006). Other studies have also confirmed that perceptive social support and positive psychological capital, which act together as an individual's internal resources and perceived environmental resources, play a mediating role between psychological factors of trauma and post-traumatic growth (Jia et al., 2015). Therefore, we propose Hypothesis 2: Positive psychological capital and perceived social support play mediating roles between family-work conflict and post-traumatic growth.

### The Moderating Role of Suppression

Some previous studies reported that the impact of family-work conflict on post-traumatic growth is not simply a stable negative predictive relationship. Gross and John (2003) proposed two mood regulation strategies: cognitive reappraisal and suppression. In their model, suppression is considered to be a reaction-centered emotional regulation strategy, which regulates emotional responses by controlling emotional expression behaviors that are about to occur or are occurring (Gross, 2010). In stressful situations, suppression may be used in an attempt to change or avoid negative thoughts and feelings (Kashdan et al., 2006). Previous studies have reported that mood regulation strategies are often important regulatory variables affecting psychological changes in task conflict situations. For example, suppression can moderate the effect of social anxiety on positive emotion (Kashdan and Breen, 2008). Similarly, we speculate that suppression moderates the impact of family-work conflict on positive psychological capital and perceived social support, affecting the improvement of post-traumatic growth.

According to the above argument and speculation, when an individual's level of suppression is high, their perceived social support and positive psychological capital levels decrease slowly with increased family-work conflict. Thus, high suppression maintains the level of perceived social support and positive psychological capital under conditions of increased family-work conflict, contributing to the realization and improvement of post-traumatic growth. Strong negative emotions are typically stimulated in family-work conflict situations, and suppression is likely to inhibit the expression of negative emotion. First, suppression strategies minimize the effects of negative stimuli by ignoring negative stimuli (e.g., family work conflicts) and avoiding paying attention to or recalling negative stimuli (Olson and Zanna, 1979). Second, suppression has a buffering function. By reducing the expression of negative emotions in stressful situations, suppression can prevent the expansion of negative emotions, playing a protective buffer role for emotions (Coifman et al., 2007). Third, regarding suppression as a target-oriented strategy, positive cognitive style has been examined using negative stimuli for a wide range of thoughts (Langens and Mrth, 2003), including a sense of mission, recognition of the importance of community, and the occurrence of family-work conflict from family concerns and care, which can be adopted as ways of helping individuals experience positive emotions and attitudes (Boden and Baumeister, 1997). When the suppression level is low, individuals do not suppress negative emotional expression. When negative emotions are experienced as overflowing, the individual experiences an increasing influence of negative emotions, which can lead to a gradual increase in stress. On the one hand, positive psychological capital and perceived social support can alleviate the individual's response to pressure. On the other hand, the individual can also be exhausted by constant stress (Zhang et al., 2013; Yang and Ye, 2014). Therefore, with increased family-work conflict, the levels of perceived social support and positive psychological capital decrease sharply, which is not conducive to the realization and improvement of post-traumatic growth. Thus, we proposed Hypothesis 3: Suppression plays a moderating role in the relationships among family-work conflict, psychological capital and social support. Besides, high suppression can buffer the negative psychological impact of family-work conflict.

## Materials and Methods

### Participants

A total of 1,347 Chinese medical workers took part in the survey, including 333 men with an average age of 35.59 years (standard deviation [SD] = 8.057, range = 20–60 years) and 1,014 women with an average age of 31.34 years (SD = 6.920, range = 20–60 years). The participants had different operational roles (doctor, medical technician, nurse, administrative logistician). Among the participants, 458 were unmarried, 871 were married, 17 were divorced, and one was widowed.

### Measures

#### Family-Work Conflict

The five-item Family-Work Conflict Scale (Netemeyer et al., 1996) was used to measure participants' family interference with work (FIW) conflict. Example items included “The demands of my family or spouse/partner interfere with work-related activities” and “Family-related strain interferes with my ability to perform job-related duties.” Participants were instructed to evaluate each statement against their own situation on a five-point Likert scale (1 = strongly disagree, 5 = strongly agree). The family-work conflict index was calculated as the total score of these five items (Cronbach's α = 0.935, Guttman split half = 0.908), with higher scores representing a greater degree of family-work conflict.

#### Post-traumatic Growth

The Post-traumatic Growth Inventory (PTGI), (Tedeschi and Calhoun, 1996) was used to assess the general tendency to experience difficult events that produce perceived benefits, focusing on various benefits that may be found or explained. The inventory contains 21 items divided into five dimensions: appreciation of life, personal strength, new possibilities, relating to others, and spiritual change (Cronbach's α = 0.959, Guttman split half = 0.928). Participants responded on a six-point scale from zero (“I did not experience this change as a result of my crisis”) to five (“I experienced this change to a very great degree as a result of my crisis”). Higher scores indicate more post-traumatic growth.

#### Positive Psychological Capital

The 26-item Positive Psychological Capital Questionnaire (PPQ), (Zhang et al., 2010) was used to measure the general positive psychological state of individuals during their growth and development (Cronbach's α = 0.908, Guttman split half = 0.847). The questionnaire was divided into four dimensions: self-efficacy (items included “My insights and abilities are above average”), resilience (items included “When I encounter setbacks, I bounce back very quickly”), hope (items included “I study and work actively to realize my dreams”) and optimism (items included “I think life is good”). Participants responded to each item on a 7-point Likert scale, with higher total scores indicating a higher level of psychological capital.

#### Perceived Social Support

Multidimensional Scale of Perceived Social Support (MSPSS), (Zimet et al., 1988) is a self-report method for subjective evaluation of social support. Twelve items were used to measure the degree to which individuals perceive support from various social support sources such as family and friends (Cronbach's α = 0.965, Guttman split half = 0.940). Items included “My family really tries to help me” and “I can talk about my problems with my friends.” Participants responded on a 7-point scale from one (strongly disagree) to seven (strongly agree). The total score of the scale represents the overall social support, and higher total scores indicated a greater degree of social support.

#### Suppression

Suppression was measured using the items of the suppression dimension of the Emotion Regulation Questionnaire (ERQ), (Gross and John, 2003), (Cronbach's α = 0.742, Guttman split half = 0.723). Four items were used to measure the emotion regulatory process by suppression strategy, which included “I control my emotions by not expressing them” and “When I am feeling negative emotions, I make sure not to express them.” Each item was scored on a 7-point Likert scale from one (strongly disagree) to seven (strongly agree). Higher scores indicated greater use of suppression strategies.

### Procedure

This research has been reviewed and approved by the Committee of Protection of Subjects (ICNL IRB) at Renmin University of China. We collected data on family-work conflict, post-traumatic growth, positive psychological capital, perceived social support, and suppression among medical workers in a city in China. The survey was conducted from October 14 to 24, 2020.

After providing informed consent, medical workers were instructed to participate in an online survey via a questionnaire platform. The survey included the family-work conflict scale, the post-traumatic growth inventory, the positive psychological capital questionnaire, the multidimensional scale of perceived social support, and the suppression dimension of the emotion regulation questionnaire. Upon completion of the online questionnaires, medical workers were asked to fill out their demographic information.

### Statistical Analysis

SPSS Statistics 21.0 software was used for statistical analyses, including correlation analyses and linear regression analyses. To further test the moderated mediation effect, the Bootstrap method was applied and Process Model 4 was used (Hayes, 2013). The mediating and the moderating effects were integrated into the same analytical framework to verify the moderated mediation model.

## Results

### Preliminary Analyses

Table 1 shows the descriptive statistics and correlations among all variables. The results revealed that post-traumatic growth was significantly negatively correlated with family-work conflict (*r* = −0.255, *p* < 0.01), partially verifying Hypothesis 1. Positive psychological capital (*r* = 0.644, *p* < 0.01) and perceived social support (*r* = 0.554, *p* < 0.01) were significantly positively correlated with post-traumatic growth to different degrees. Family-work conflict was negatively correlated with positive psychological capital (*r* = −0.405, *p* < 0.01) and perceived social support (*r* = −0.407, *p* < 0.01). There was no significant correlation between suppression and family-work conflict (*r* = 0.01, *p* = 0.722), indicating that the two factors were independent.

**Table 1:** Means, standard deviations, and correlation matrix among all variables.

***Variables***	***Mean***	***SD***	***1***	***2***	***3***	***4***
1. Family-work conflict	11.04	4.388				
2. Post-traumatic growth	81.81	19.547	−0.255^**^			
3. Positive psychological capital	86.21	15.000	−0.405^**^	0.644^**^		
4. Perceived social support	43.17	9.044	−0.407^**^	0.554^**^	0.648^**^	
5. Suppression	12.26	2.663	0.010	0.167^**^	0.144^**^	0.141^**^

** *p < 0.01*.

### The Mediate Effect of Positive Psychological Capital and Perceived Social Support

In linear regression, family-work conflict negatively predicted post-traumatic growth (*B* = −1.136, 95% CI [−1.367, −0.906], *SE* = 0.117, *t* = −9.676, *p* < 0.001). To examine whether positive psychological capital and perceived social support mediated the correlation between family-work conflict and post-traumatic growth, the Bootstrap method was applied, and Process Model 4 was used (Hayes, 2013). The results indicated that family-work conflict negatively predicted positive psychological capital (*B* = −1.386, 95% CI [−1.553, −1.219], *SE* = 0.085, *t* = −16.266, *p* < 0.001) and perceived social support (*B* = −0.839, 95% CI [−0.940, −0.739], *SE* = 0.051, *t* = −16.350, *p* < 0.001). Family-work conflict (*B* = 0.223, 95% CI [0.025, 0.421], *SE* = 0.101, *t* = 2.207, *p* = 0.0275), positive psychological capital (*B* = 0.657, 95% CI [0.587, 0.726], *SE* = 0.035, *t* = 18.543, *p* < 0.001) and perceived social support (*B* = 0.535, 95% CI [0.420, 0.651], *SE* = 0.059, *t* = 9.108, *p* < 0.001) positively predicted post-traumatic growth.

**Figure 1:**
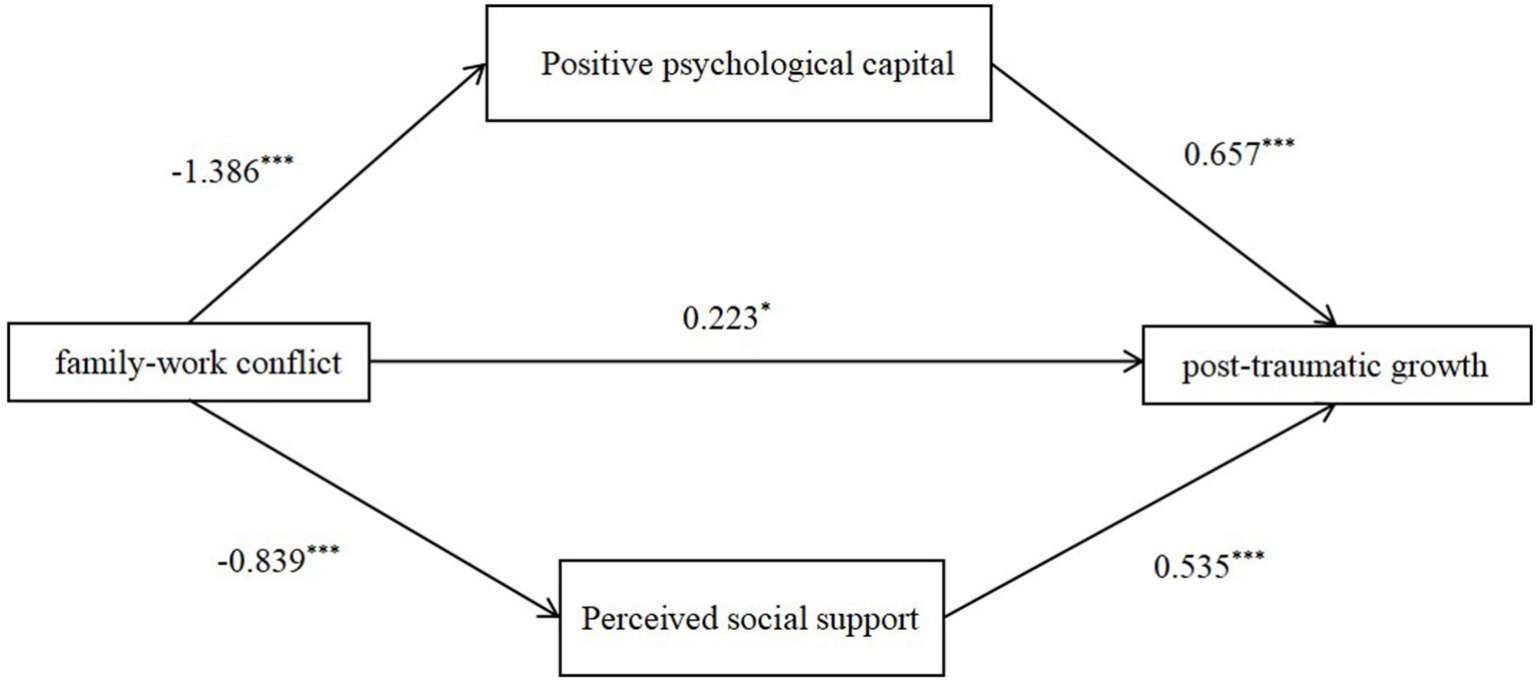
The mediating effect of positive psychological capital and perceived social support. *p < *0.05*, ***p < 0.001.

Further analysis revealed that positive psychological capital and perceived social support played mediating roles in the path from family-work conflict to post-traumatic growth (see Figure 1). The Bootstrap method was used to test the significance of mediating effects with 1,000 Bootstrap samples (Preacher and Hayes, 2008). The results revealed that family-work conflict had a significant indirect effect on post-traumatic growth through positive psychological capital (*B* = −0.910, 95% CI [−1.090, −0.732], *SE* = 0.092). Family-work conflict had a significant indirect effect on post-traumatic growth through perceived social support (*B* = −0.450, 95% CI [−0.600, −0.322], *SE* = 0.073), (see Table 2). Thus, Hypothesis 2 was supported.

**Table 2:** Test results of mediating effect analysis.

	***B***	***SE***	***95% CI***	
			***Lower***	***Upper***
Positive psychological capital	−0.910	0.092	−1.090	−0.732
Perceived social support	−0.450	0.073	−0.600	−0.322
Total	−1.359	0.113	−1.581	−1.130

### The Moderate Effect of Suppression

**Figure 2:**
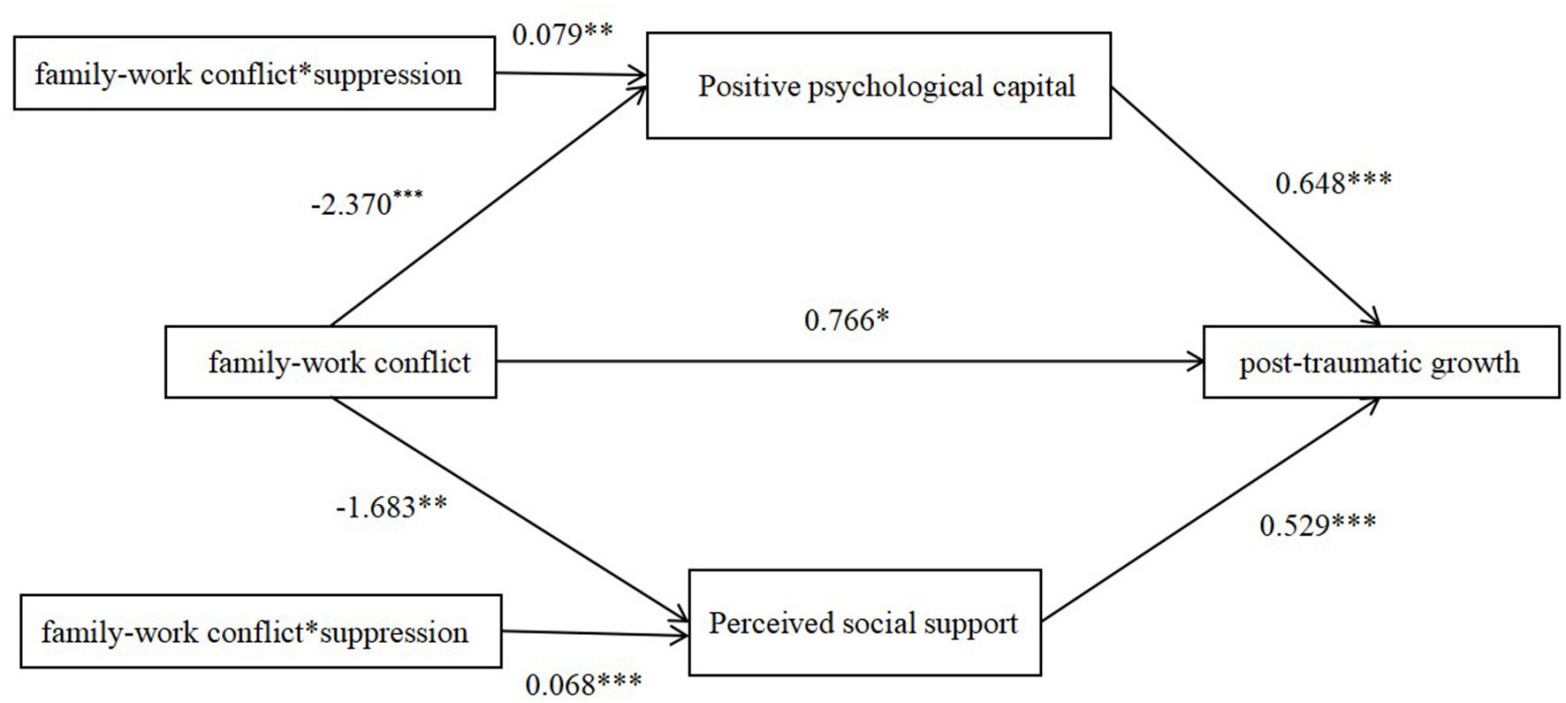
The moderate effect of suppression on the mediating effect of positive psychological capital and perceived social support. *p < *0.05*, **p < *0.01*, ***p < 0.001.

The results revealed that the interaction between family-work conflict and suppression had no significant predictive effect on post-traumatic growth (*B* = −0.045, 95% CI = [−0.102, 0.011]). The interaction terms of family-work conflict and suppression had significant positive predictive effects on positive psychological capital (*B* = 0.079, 95% CI [0.027, 0.131], *SE* = 0.027, *t* = 2.96, *p* = 0.003) and perceived social support (*B* = 0.068, 95% CI [0.036, 0.099], *SE* = 0.016, *t* = 4.223, *p* < 0.001), (see Figure 2). The regression coefficients of the three models are presented in Table 3.

**Table 3:** Model for the moderated-mediation hypothesis.

***Model***	***Dependent variable***	***Independent*** ***variable***	***R^2^***	***B***	***p***	***SE***	***t***	***95% CI***	
								***Lower***	***Upper***
1	M1	X	0.192	−2.370^***^	0.000	0.342	−6.935	−3.041	−1.700
		W		0.041	0.892	0.302	0.135	−0.552	0.634
		X*W		0.079^**^	0.003	0.027	2.96	0.027	0.131
2	M2	X	0.198	−1.683^***^	0.000	0.205	−8.195	−2.085	−1.280
		W		−0.188	0.299	0.181	−1.038	−0.544	0.168
		X*W		0.068^***^	0.000	0.016	4.223	0.036	0.099
3	Y	X	0.453	0.766^*^	0.042	0.377	2.034^*^	0.027	1.505
		M1		0.648^***^	0.000	0.035	18.304	0.579	0.718
		M2		0.529^***^	0.000	0.059	8.970	0.413	0.645
		W		0.898^**^	0.006	0.324	2.770	0.262	1.534
		X*W		−0.045	0.116	0.029	−1.572	−0.102	0.011

* *p < 0.05*,

** *p < 0.01*,

*** *p < 0.001. M1, positive psychological capital; M2, perceive social support; X, family-work conflict; Y, post-traumatic growth; W, suppression*.

**Figure 3:**
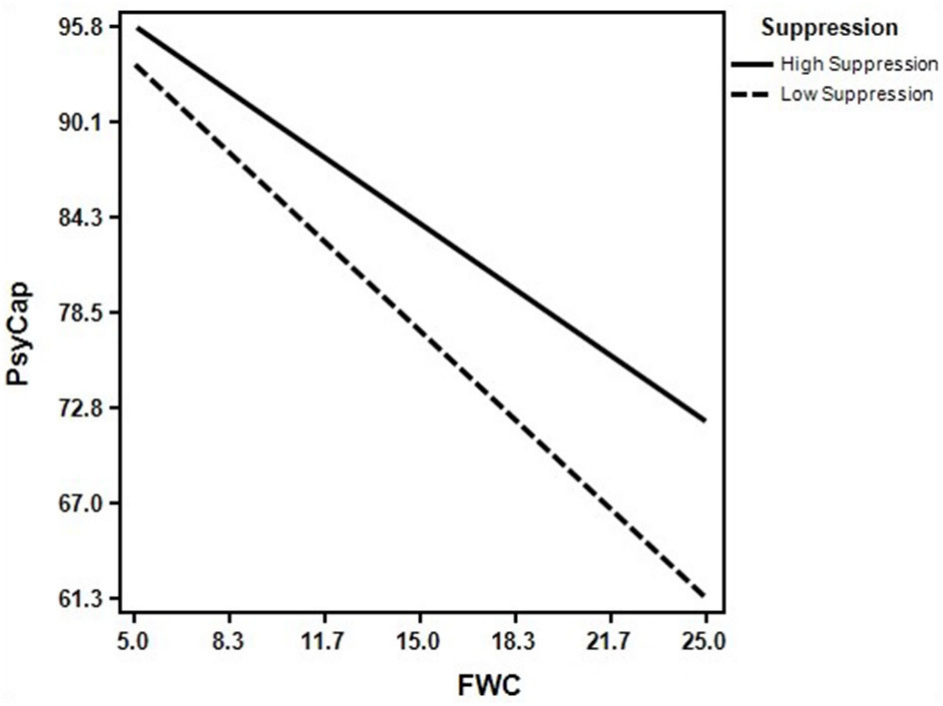
The moderating effect of suppression on family-work conflict to positive psychological capital.

To further analyze the moderating effect of suppression, we constructed a moderating effects diagram of suppression. When participants exhibited high suppression (+1 SD from the mean), the mediating effect of positive psychological capital (*B* = −1.046, 95% CI [−1.312, −0.833]) was lower than that for participants with low suppression (−1 SD from the mean), (*B* = −0.774, 95% CI [−0.994, −0.597]). The results revealed that high suppression can buffer the negative effect of family-work conflict on positive psychological capital (see Table 4 and Figure 3).

**Table 4:** Mediating effect values of positive psychological capital and perceived social support on different levels of suppression.

**Variables**	**Suppression level**	***B***	***SE***	***95%CI***	
				***Lower***	***Upper***
Positive psychological capital	9.593	−1.046	0.124	−1.312	−0.833
	14.918	−0.774	0.104	−0.994	−0.597
Perceived social support	9.593	−0.547	0.089	−0.768	−0.397
	14.918	−0.356	0.069	−0.511	−0.235

**Figure 4:**
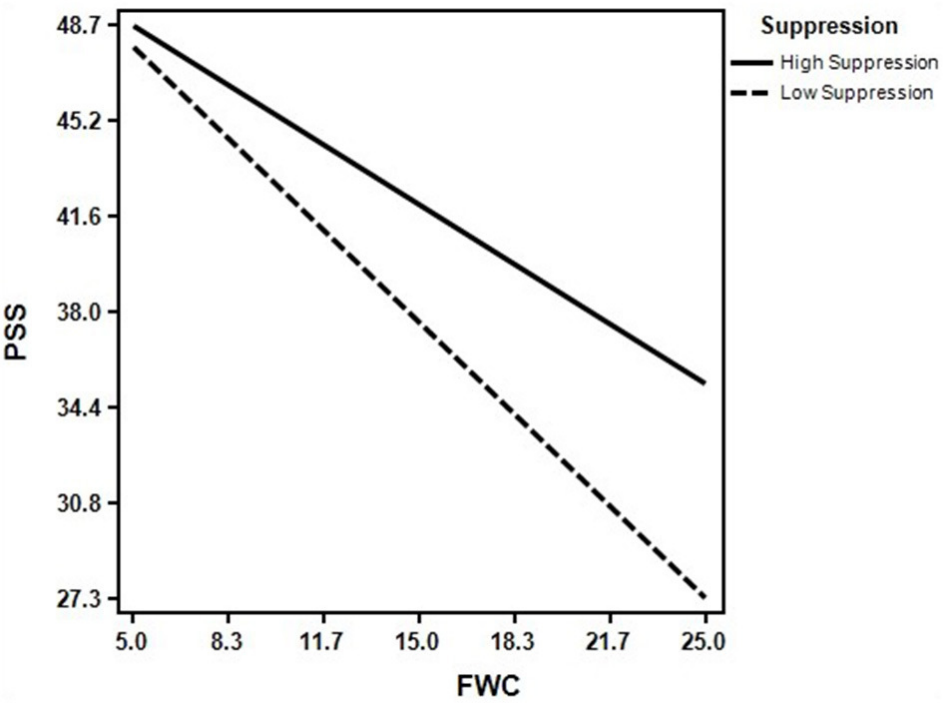
The moderating effect of suppression on family-work conflict to perceived social support.

The analysis of results shown in Table 4 and Figure 4 shows that the mediating effect of perceived social support under conditions of high suppression (*B* = −0.356, 95% CI [−0.511, −0.235]) was lower than that under conditions of low suppression (*B* = −0.547, 95% CI [−0.768, −0.397]). The results revealed that high suppression can buffer the negative effect of family-work conflict on perceived social support. Thus, Hypothesis 3 was verified.

## Discussion

The current study examined the roles of positive psychological capital, perceived social support, and suppression in family-work conflict and post-traumatic growth among medical workers during the COVID-19 pandemic. The results revealed that family-work conflict negatively predicted post-traumatic growth, and reducing family-work conflict effectively enhanced post-traumatic growth. Furthermore, positive psychological capital and perceived social support were found to play a mediating role in this process, and family-work conflict decreased positive psychological capital and perceived social support, further reducing post-traumatic growth. Moreover, suppression played a moderating role in this process, and a high level of buffer the harm of family-work conflict to positive psychological capital and perceived social support. With a high level of suppression, the negative effects of family-work conflict on positive psychological capital and perceived social support were reduced, thus reducing the negative effect of family-work conflict on post-traumatic growth.

The most important implication of the current findings is that, under the severe pressure of COVID-19 prevention and control, and the reality of substantial conflicts between family and work, medical workers with low levels of suppression in a state of constantly heightened, constantly to express their emotional expression were less able to resolve practical difficulties; rather, because negative emotions were experienced as overflowing, make it continue in the negative or pessimistic thoughts (Kim et al., 1998). A high degree of suppression can significantly alleviate the negative effects of family-work conflict on positive psychological capital and perceived social support (Zhou and Wang, 2012). These findings suggest that, when medical workers are subjected to severe family-work conflicts, efforts to restrain the expression of emotions can help maintain their level of positive psychological capital and perceived social support, thus improving post-traumatic growth. Although this result conflicts with a previous report that suppression is associated with low self-esteem and is detrimental to establishing intimate relationships with others and thus to individual growth (Farmer and Kashdan, 2012), it is consistent with the conclusions of previous studies in some stress or disaster scenarios. For example, some studies have found that the transformation of post-traumatic stress disorder to post-traumatic growth leads to more emotional expression suppression strategies (Tian et al., 2016). In the current study, this may have occurred because medical workers who had experienced pandemic trauma were still experiencing high family-work conflict. By adopting a high level of suppression strategies, individuals may have been able to buffer the negative consequences of family-work conflicts, effectively protecting their internal positive states and external support resources.

Moreover, our results also suggest that the applicability of suppression strategies can be discussed in a wider range of applications, and may be related to environmental features (Aldao, 2013), particularly cultural factors. A previous study reported that, in countries with more individualistic cultures, the role of suppression is typically negative, whereas in countries with more collectivist cultures, suppression tends to have positive influences on emotional experience, interpersonal relationships, and psychological and social adaptation, which can weaken or even reverse negative relationships with positive outcome variables (Liu et al., 2016). This finding was supported by the current results. In collectivist cultural contexts, individuals are often required to exhibit restraint and remain calm (Soto et al., 2011), and self-dependence involves a tendency to adopt inhibition strategies to achieve interpersonal harmony (Yang and Lu, 2009). Therefore, high levels of suppression may be encouraged and praised. If this behavior persists over a long period of time, the stress state can become aggravated. Thus, in this continuous stress state, the individual's positive psychological capital and perceived social support may also be damaged, which is not conducive to the realization of post-traumatic growth. In the face of pandemic trauma and objectively intensified family-work conflict, medical workers controlling emotions by suppressing emotional expression, avoiding emotional expression in positive or negative mood states, and hiding emotions can effectively alleviate family-work conflict. In turn, this process can promote self-efficacy, resilience, hope, and optimism among medical workers, helping to maintain the feeling that there are others around to share happiness and sorrow with and who can offer help and support, which facilitates the maintenance of good interpersonal relationships. Further investigation of cross-cultural dynamics and the complexity of this model will be required to elucidate these issues in more detail. Consistent with previous cross-cultural studies, the current findings support the positive effects of suppression, which is consistent with previous cross-cultural studies and extending the findings of previous studies of suppression.

The current results also revealed negative effects and path mechanisms of family-work conflict on post-traumatic growth. The results revealed that positive psychological capital and perceived social support played mediating roles in the negative impact of family-work conflicts on post-traumatic growth. This conclusion fits with the logic of the J-DR model and COR theory. Family-work conflict and work requirements can impair positive psychological capital, perceived social support, and other work resources, which is not conducive to the growth and development of the individual. Furthermore, individuals can fully utilize personal resources (such as positive psychological capital), and, with the help of external factors (such as perceived social support), cope with high-pressure environments to achieve good outcomes (Hobfoll, 2002).

The current study has several limitations, and the findings should be treated with caution. First, the survey was conducted ~6 months after the outbreak of pandemic, and, as pandemic developed, the various conditions and performances of medical workers may have changed accordingly. Thus, it may be useful to further track the study population and extend the current findings. Second, the current study only involved academic research, and interventions with medical workers have not yet been conducted to promote appropriate psychological adjustment based on the relevant conclusions of this study. Third, the current study only used self-report measures, and did not assess personality traits involved in post-traumatic growth and suppression strategies. In the future, a variety of assessment methods can be added, and the corresponding influencing factors can be added to make the research results more effective.

Therefore, based on the current findings in the context of the COVID-19 pandemic, we propose the following recommendations: first, medical workers should be encouraged to use methods to avoid family-work conflict, reduce negative emotion expansion, and enhance positive cognition, and effectively use suppression strategies to enhance their individual internal strength and external resources; second, rather than focusing excessive attention on medical workers themselves, we should avoid exacerbating the pressure they experience and depleting their psychological resources and should direct more care to the families of medical workers, helping to effectively resolve difficulties and needs in the family.

## Data Availability Statement

The raw data supporting the conclusions of this article will be made available by the authors, without undue reservation.

## Ethics Statement

The studies involving human participants were reviewed and approved by Committee of Protection of Subjects (ICNL IRB) at Renmin University of China. The patients/participants provided their written informed consent to participate in this study.

## Author Contributions

ML and XT contributed to all aspects of work for this article. CX contributed to conception, and design and revising the article critically. JZ contributed to data analysis and revising the article critically. SH contributed to revising the article critically. All authors contributed to the article and approved the submitted version.

## Funding

This research is supported by the granting agency: Building World-Class Universities (disciplines) of Renmin University of China (RUCPSY0008).

## Conflict of Interest

The authors declare that the research was conducted in the absence of any commercial or financial relationships that could be construed as a potential conflict of interest.

## Publisher's Note

All claims expressed in this article are solely those of the authors and do not necessarily represent those of their affiliated organizations, or those of the publisher, the editors and the reviewers. Any product that may be evaluated in this article, or claim that may be made by its manufacturer, is not guaranteed or endorsed by the publisher.

## References

[B1] Aldao A. (2013). The future of emotion regulation research capturing context. Perspect. Psychol. Sci. 8, 155–172. 10.1177/1745691612459518 26172497

[B2] Bakker A. B. Demerouti E. (2017). Job demands-resources theory: taking stock and looking forward. J. Occup. Health Psychol. 22, 273–285. 10.1037/ocp0000056 27732008

[B3] Boden J. M. Baumeister R. F. (1997). Repressive coping: distraction using pleasant thoughts and memories. J. Pers. Soc. Psychol. 73, 45–62. 10.1037/0022-3514.73.1.45 9216078

[B4] Casellas-Grau A. Ochoa C. Ruini C. (2017). Psychological and clinical correlates of post-traumatic growth in cancer. A systematic and critical review. Psycho-Oncol. 26, 2007–2018. 10.1002/pon.4426 28317221

[B5] Chen X. Li Q. Xu F. Han B. (2021). The mediating role of resilience between work–family conflict and career development among Chinese nurses: a cross-sectional study. J. Nurs. Manag. 1. 10.1111/jonm.13323 33797812

[B6] Cohen S. Wills T. A. (1985). Stress, social support, and the buffering hypothesis. Psychol. Bull. 98, 310–357. 10.1037/0033-2909.98.2.310 3901065

[B7] Coifman K. G. Bonanno G. A. Ray R. D. Gross J. J. (2007). Does repressive coping promote resilience? Affective-autonomic response discrepancy during bereavement. J. Pers. Soc. Psychol. 92, 745–758. 10.1037/0022-3514.92.4.745 17469956

[B8] Farmer A. S. Kashdan T. B. (2012). Social anxiety and emotion regulation in daily life: spillover effects on positive and negative social events. Cogn. Behav. Ther. 41, 152–162. 10.1080/16506073.2012.666561 22428662 PMC3370054

[B9] Gross J. (2010). Emotion regulation: affective, cognitive, and social consequences. Psychophysiology 39, 281–291. 10.1017/S0048577201393198 12212647

[B10] Gross J. J. John O. P. (2003). Individual differences in two emotion regulation processes: implications for affect, relationships, and well-being. J. Pers. Soc. Psychol. 85, 348–362. 10.1037/0022-3514.85.2.348 12916575

[B11] Hayes A. F. (2013). Introduction to mediation, moderation, and conditional process analysis: a regression-based approach. J. Educ. Meas. 51, 335–337. 10.1111/jedm.12050

[B12] Hobfoll S. E. (1989). Conservation of resources: a new attempt at conceptualizing stress. Am. Psychol. 44, 513–524. 10.1037/0003-066X.44.3.513 2648906

[B13] Hobfoll S. E. (2002). Social and psychological resources and adaptation. Rev. Gen. Psychol. 6, 307–324. 10.1037/1089-2680.6.4.307

[B14] Jia X. Ying L. Zhou X. Wu X. Lin C. (2015). The effects of extraversion, social support on the post-traumatic stress disorder and post-traumatic growth of adolescent survivors of the Wenchuan earthquake. PLoS ONE 10:e0121480. 10.1371/journal.pone.0121480 25815720 PMC4376870

[B15] Kang L. J. Li Y. Hu S. H. Chen M. Yang C. Yang B. X.. (2020). The mental health of medical worker in Wuhan, China dealing with the 2019 novel coronavirus. Lancet Psychiatry 7:e14. 10.1016/S2215-0366(20)30047-X 32035030 PMC7129673

[B16] Kashdan T. B. Barrios V. Forsyth J. P. Steger M. F. (2006). Experiential avoidance as a generalized psychological vulnerability: comparisons with coping and emotion regulation strategies. Behav. Res. Ther. 44, 1301–1320. 10.1016/j.brat.2005.10.003 16321362

[B17] Kashdan T. B. Breen W. E. (2008). Social anxiety and positive emotions: a prospective examination of a self-regulatory model with tendencies to suppress or express emotions as a moderating variable. Behav. Ther. 39, 1–12. 10.1016/j.beth.2007.02.003 18328865

[B18] Kim D. Pan Y. Park H. S. (1998). High-vs. low-context culture: a comparison of Chinese, Korean, and American cultures. Psychol. Mark. 15, 507–521. 10.1002/(SICI)1520-6793(199809)15:6<507::AID-MAR2>3.0.CO;2-A

[B19] Langens T. A. Mrth S. (2003). Repressive coping and the use of passive and active coping strategies. Pers. Individ. Dif. 35, 461–473. 10.1016/S0191-8869(02)00207-6

[B20] Liu Y. Sang B. Gong S. Y. Ding X. C. Pan T. T. (2016). Cultural differences on function of emotional expression suppression. Adv. Psychol. Sci. 24, 1647–1654. 10.3724/SP.J.1042.2016.01647

[B21] López-Núez M. I. Díaz-Morales J. F. Aparicio-García M. E. (2021). Individual differences, personality, social, family and work variables on mental health during COVID-19 outbreak in Spain. Pers. Individ. Dif. 172:110562. 10.1016/j.paid.2020.110562

[B22] Luthans F. Youssef-Morgan C. M. Avolio B. J. (2007). Psychological capital: developing the human competitive edge. Oxford, NY: Oxford University Press.

[B23] Netemeyer R. G. Boles J. S. Mcmurrian R. (1996). Development and validation of work-family conflict and family-work conflict scales. J. Appl. Psychol. 81, 400–410. 10.1037/0021-9010.81.4.400

[B24] Olson J. M. Zanna M. P. (1979). A new look at selective exposure. J. Exp. Soc. Psychol. 15, 1–15. 10.1016/0022-1031(79)90014-3

[B25] Preacher K. J. Hayes A. F. (2008). Asymptotic and resampling strategies for assessing and comparing indirect effects in multiple mediator models. Behav. Res. Methods 40, 879–891. 10.3758/BRM.40.3.879 18697684

[B26] Reff R. C. Kwon P. Campbell D. G. (2005). Dysphoric responses to a naturalistic stressor: Interactive effects of hope and defense style. J. Soc. Clin. Psychol. 24, 638–648. 10.1521/jscp.2005.24.5.638

[B27] Schaefer J. Moos R. (1998). The context for post-traumatic growth: life crises, individual and social resources, and coping., in Post-traumatic Growth: Positive Changes in the Aftermath of Crisis, eds Tedeschi R. Park C. Calhoun L. (Mahwah, NJ: Erlbaum), 99-126.

[B28] Shand L. K. Cowlishaw S. Brooker J. E. Burney S. Ricciardelli L. A. (2015). Correlates of post-traumatic stress symptoms and growth in cancer patients: a systematic review and meta-analysis. Psycho-Oncol. 24, 624–634. 10.1002/pon.3719 25393527

[B29] Sheikh A. I. (2008). Post-traumatic growth in trauma survivors: implications for practice. Couns. Psychol. Q. 21, 85–97. 10.1080/09515070801896186

[B30] Soto J. A. Perez C. R. Kim Y. H. Lee E. A. Minnick M. R. (2011). Is expressive suppression always associated with poorer psychological functioning? A cross-cultural comparison between European Americans and Hong Kong Chinese. Emotion 11, 1450–1455. 10.1037/a0023340 21707152

[B31] Tedeschi R. G. Calhoun L. G. (1995). xTrauma & transformation: Growing in the aftermath of suffering. Sage Publications, Inc.

[B32] Tedeschi R. G. Calhoun L. G. (1996). The post-traumatic growth inventory: measuring the positive legacy of trauma. J. Trauma. Stress 9, 455–471. 10.1002/jts.2490090305 8827649

[B33] Tedeschi R. G. Calhoun L. G. (2004). Post-traumatic growth: conceptual foundations and empirical evidence. Psychol. Inq. 15, 1–18. 10.1207/s15327965pli1501_01

[B34] Tedeschi R. G. Park C. L. Calhoun L. G. (1998). Post-traumatic growth: conceptual issues, in Post-Traumatic Growth: Positive Changes in the Aftermath of Crisis, eds Tedeschi R. G. Park C. L. Calhoun L. G. (Mahwah, NJ: Erlbaum), 99-126. 10.4324/9781410603401

[B35] Tian Y. X. Zhou X. Wu X. C. Zeng M. (2016). The moderating role of emotion regulation between PTSD and PTG. Chinese J. Clin. Psychol. 24, 480-483. 10.16128/j.cnki.1005-3611.2016.03.021

[B36] Vaziri H. Casper W. J. Wayne J. H. Matthews R. A. (2020). Changes to the work-family interface during the COVID-19 pandemic: examining predictors and implications using latent transition analysis. J. Appl. Psychol. 105, 1073–1087. 10.1037/apl0000819 32866024

[B37] Yang K. S. Lu L. (2009). The Chinese Self: Psychological Analysis. Honolulu: National Taiwan University Press.

[B38] Yang Q. Ye B. J. (2014). Stressful life events on drug use among reform school students: the effect of family functioning and perceived social support. J. Psychol. Sci. 37, 111–116. 10.16719/j.cnki.1671-6981.2014.01.021

[B39] Zhang K. Zhang S. Dong Y. H. (2010). Positive psychological capital: Measurement and its relationship with mental health. Stud. Psychol. Behav. 8, 58–64. 10.3969/j.issn.1672-0628.2010.01.01112656956

[B40] Zhang K. Zou Y. Wang X. J. (2013). The role of psychological capital between job stressors and stress responses: Mediator or moderator? Psychol. Explor. 6, 53–57. 10.3969/j.issn.1003-5184.2013.06.009

[B41] Zhang W. R. Wang K. Yin L. Zhao W. F. Wang H. X. (2020). Mental health and psychosocial problems of medical health workers during the COVID-19 epidemic in China. Psychother. Psychosom. 89, 1–9. 10.1159/000507639 32272480 PMC7206349

[B42] Zhou T. Wang D. F. (2012). Usage of emotion suppression and its relationship with mental health. Chinese J. Clin. Psychol. 20, 65-68, 64. 10.16128/j.cnki.1005-3611.2012.01.027

[B43] Zimet G. D. Dahlem N. W. Zimet S. G.. (1988). The multidimensional scale of perceived social support. J. Pers. Assess. 52, 30–41. 10.1207/s15327752jpa5201_2

[B44] Zoellner T. Maercker A. (2006). Post-traumatic growth in clinical psychology–a critical review and introduction of a two component model. Clin. Psychol. Rev. 26, 626–653. 10.1016/j.cpr.2006.01.008 16515831

